# Factors influencing heterogeneity of radiation-induced DNA-damage measured by the alkaline comet assay

**DOI:** 10.1186/1748-717X-7-61

**Published:** 2012-04-20

**Authors:** Clemens Seidel, Christine Lautenschläger, Jürgen Dunst, Arndt-Christian Müller

**Affiliations:** 1 Department of General Psychiatry I, PZN-Wiesloch, Teaching Hospital of Ruprecht-Karls-University Heidelberg, Heidelberger Str. 1a, Wiesloch, 69168, Germany; 2 Institute of Biostatistics, Martin-Luther-University, Magdeburger Straße 8, Halle, 06112, Germany; 3 Department of Radiotherapy, University of Lübeck, Ratzeburger Allee 160, Lübeck, 23538, Germany; 4 Department of Radiooncology, Eberhard-Karls-University, Hoppe-Seyler-Strasse 3, Tübingen, Germany

**Keywords:** Heterogeneity, Comet assay, %Tail DNA, Antioxidants, Histones

## Abstract

**Background:**

To investigate whether different conditions of DNA structure and radiation treatment could modify heterogeneity of response. Additionally to study variance as a potential parameter of heterogeneity for radiosensitivity testing.

**Methods:**

Two-hundred leukocytes per sample of healthy donors were split into four groups. I: Intact chromatin structure; II: Nucleoids of histone-depleted DNA; III: Nucleoids of histone-depleted DNA with 90 mM DMSO as antioxidant. Response to single (I-III) and twice (IV) irradiation with 4 Gy and repair kinetics were evaluated using %Tail-DNA. Heterogeneity of DNA damage was determined by calculation of variance of DNA-damage (V) and mean variance (Mvar), mutual comparisons were done by one-way analysis of variance (ANOVA).

**Results:**

Heterogeneity of initial DNA-damage (I, 0 min repair) increased without histones (II). Absence of histones was balanced by addition of antioxidants (III). Repair reduced heterogeneity of all samples (with and without irradiation). However double irradiation plus repair led to a higher level of heterogeneity distinguishable from single irradiation and repair in intact cells. Increase of mean DNA damage was associated with a similarly elevated variance of DNA damage (r = +0.88).

**Conclusions:**

Heterogeneity of DNA-damage can be modified by histone level, antioxidant concentration, repair and radiation dose and was positively correlated with DNA damage. Experimental conditions might be optimized by reducing scatter of comet assay data by repair and antioxidants, potentially allowing better discrimination of small differences. Amount of heterogeneity measured by variance might be an additional useful parameter to characterize radiosensitivity.

## Introduction

Reliable determination of radiosensitivity is of great importance in radiation oncology [1]. However, intra-individual heterogeneity and inter-individual variability of radiation-induced DNA damage limit test sensitivity, particularly in cases of small differences in radiosensitivity. Variability can be reduced by a highly standardized operation protocol and human reference sample [2]. Regarding heterogeneity, there are some assumptions relating this phenomenon to functionally radio-resistant subpopulations [3,4]. Despite recent advances, a clear differentiation of radio-resistant subgroups within a cell line by cell surface markers or other criteria is not possible [5].

To study reasons for and the extent of heterogeneity, we searched for a cell model with an almost homogeneous response to irradiation to attribute changes of distribution to heterogeneity-inducing factors more easily. Radio-sensitive cells without extreme differences in response to irradiation appeared appropriate for this. We therefore used leukocytes from healthy volunteers to investigate how little intrinsic heterogeneity [6,7] might be influenced by changes in DNA conformation, antioxidant level and different radiation schedules. Radiation-induced DNA damage was measured by single cell gel electrophoresis (SCGE, also called comet assay) initially established by Östling and Johannson [8] capable in its alkaline modification of detecting single strand breaks (SSB), alkali labile sites and incomplete excision repair sites [9-11]. We defined heterogeneity as variance of DNA damage since this parameter was usually applied to describe the scattering of data.

The aim of the present study was to evaluate whether heterogeneity could be modified by treatment related (radiotherapy and repair) or structural conditions (radical scavenging property, DNA organisation). Furthermore, the present study investigated whether variance as descriptor of heterogeneity could become a useful parameter to give additional aspects of radiation sensitivity.

## Material and methods

Chemicals were purchased from the following suppliers: Agarose, low melting point (LMP) agarose and phosphate-buffered saline (PBS) from Gibco BRL, Paisley, UK; RPMI 1640 (with 25 mM Hepes and L-Glutamine) from Biowhittaker Europe, Verviers, Belgium; fetal calf serum from Biochrom AG, Berlin, Germany; Sodium chloride from Roth, Karlsruhe, Germany; EDTA, Triton X-100 and propidium iodide from Sigma, Deisenhofen, Germany; Tris(hydroxymethylaminomethane), DMSO, sodium hydroxide and hydrochloric acid from Merck, Darmstadt, Germany.

Blood samples were collected in citrate and EDTA tubes from five healthy volunteers (informed consent was obtained) with the following characteristics: Young age (20–25 years), no exhaustive physical activity, no smoking history, no acute or chronic disease, no vegetarian eating habits, no vitamin supplements and no medication except for contraceptives. Blood cell counts (EDTA-tubes) were taken to ensure leukocyte levels were in the reference range. Each citrate blood sample was split into several fractions for separate treatment to evaluate different conditions of DNA-organisation.

The alkaline technique described by Singh et al. [11,12] was used with some modifications [13]. In brief, experiments were performed in sandwichlayer-technique on fully frosted slides which were pre-coated with a temporary agarose layer (1000 μl, 1% in PBS). Single cells (25.000-40.000 cells per slide) were embedded in LMP-agarose (85 μl, 0.5%) with RPMI 1640 above a layer consisting of agarose (300 μl, 0.6% in PBS). Subsequently the slides were covered by a top layer of LMP-agarose with RPMI 1640.

**Figure 1:**
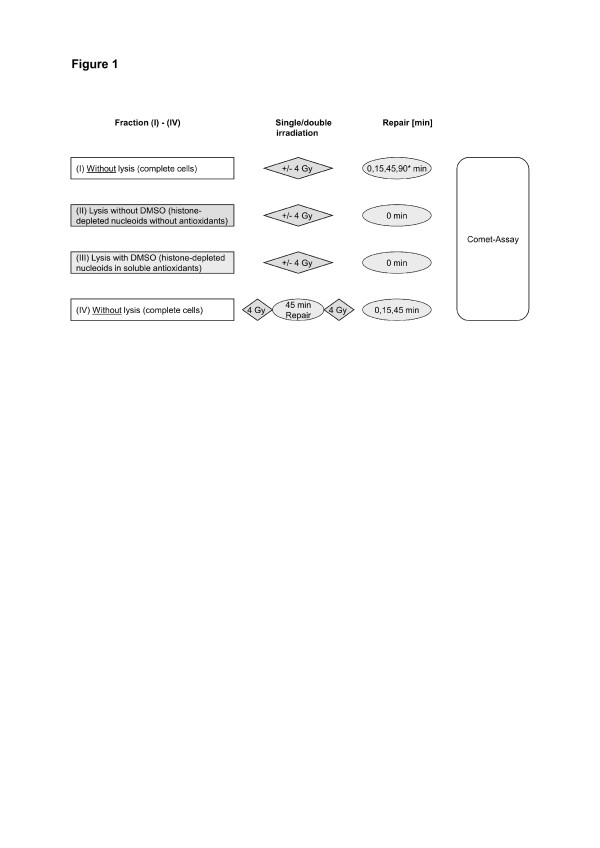
**Experimental model.** Fractions characterized by different conditions of DNA structure, antioxidant status or different radiation and repair schedules. Intact cells represent complete cells with unchanged chromatin structure or intracellular radical scavengers (Fraction I/IV). Pre-treatment lysis of fraction (II) and (III) depleted histones and radical scavengers. Therefore, fraction (II) and (III) contained DNA stripped of histones +/− antioxidant DMSO. All samples were irradiated with 4 Gy. Fraction (IV) was twice irradiated with 4 Gy after 45 min of repair time. The experimental model was examined using 200 leukocytes/sample of each donor (n = 5).

In a first set of experiments (200 leukocytes/sample of one donor), increasing radiation doses of 0-8 Gy were used to test the sensitivity of comet assay for subpopulations to define optimal radiation dose. Thereafter, three different conditions of DNA-organisation or antioxidant level were investigated in leukocytes, later referred to as fraction I-III (Figure 1). Fraction I contained unchanged cells with complete chromatin structure and antioxidants, i.e. control cells. Fraction II was lysed at 4°C for 60 min in a solution of 1.5 M sodium chloride and 1% Triton-X 100 to develop histone-depleted DNA anchored to nuclear matrix [14]. Fraction III consisted of almost pure DNA of fraction II supplemented by antioxidants (90 mM DMSO), approximately equal to intracellular scavenging capacity [15]. Irradiation of slides (fraction I-III) was performed between piacryl plates using a Philips RT 250 (200 kV, dose rate of 208.3 cGy/min). To evaluate whether double fractionated irradiation could be usable as discriminator of individual heterogeneity, we irradiated untreated cells (fraction IV) after a repair period of 45 min again, Figure 1. The time interval between both radiation treatments was based on previous repair investigations showing that 45 min were sufficient to reach basal level of DNA damage. For DNA repair studies, the cells of fraction I and IV were incubated for 0,15 or 45 min in complete medium (85% RPMI 1640, 15% fetal calf serum) at 37°C.

The slides were transferred to alkaline lysis solution (2.5 M sodium chloride, 90 mM EDTA, 10 mM Tris, 190 mM sodium hydroxide, 1% Triton-X 100 and 90 mM DMSO, pH = 10; T = 4°C) for 3 h. Alkaline unwinding (1 h) and horizontal gel electrophoresis (0.8 V/cm, 25 V, 300 mA, 30 min) were subsequently performed in the same electrophoretic buffer (2 mM EDTA, 0.6 M NaOH; pH ≥ 13.3; T = 12-14°C). After 12 h of neutralisation in 0.4 M Tris–HCl (pH = 7.5; T = 4°C) all slides were stained with 100 μl of 1% propidium iodide. DNA damage was determined by a Zeiss fluorescence microscope with a CCD video camera (Pulnix-765E, Kinetic Imaging) and an image processing system (Comet 3.1®-Kinetic Imaging). At least duplicate slides were evaluated for each donor (n = 5). Two-hundred cells per fraction for each donor were analyzed using %Tail-DNA to describe DNA-damage. Basal damage was defined as damage without irradiation. Initial damage was directly assessed after irradiation (0 min repair time). Residual damage represented the remaining damage after repair time.

Heterogeneity of DNA damage was evaluated by variance (V) of DNA damage in %Tail-DNA, for each fraction (5 samples, i.e. 1.000 comet measurements) mean variance (M_var_) was determined. Mutual comparisons between fractions were performed by one-way analysis of variance (ANOVA) accepting a p-value below 0.05 for significance. Statistical analysis, diagram, histogram plots, regression analysis and Pearson’s correlation were performed with SPSS 10.0 (Software, SPSS Inc.) and Microsoft Office (Software, Microsoft Corp.).

## Results

### Methodological considerations

**Figure 2:**
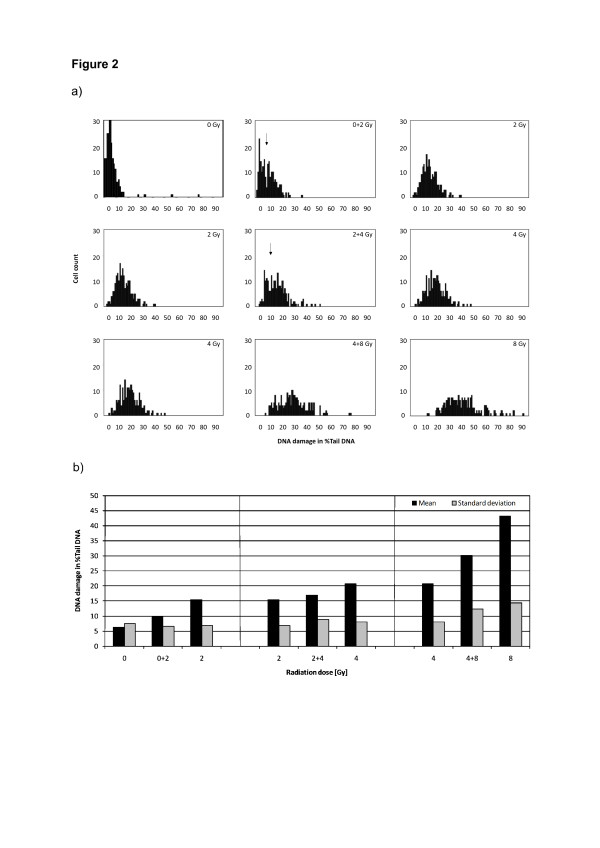
**Discrimination of heterogeneity with the alkaline Comet assay.** The sensitivity of the comet assay for subpopulations was investigated. Therefore, samples treated with 0, 2, 4 or 8 Gy and cell mixtures (0 and 2 Gy, 2 and 4 Gy, 4 and 8 Gy) were analysed (200 leukocytes/sample of one donor). The respective histograms **(a)** and DNA damage in %Tail DNA with the corresponding standard deviations **(b)** were demonstrated. Two populations were detectable for mixture-samples from 0 to 4 Gy as indicated by a subdividing arrow.

Before testing of different DNA-conditions, distribution of DNA damage at increasing radiation doses was investigated to define the optimal radiation dose for evaluation of heterogeneity. DNA-damage distribution of one donor was therefore evaluated in a histogram plot, Figure 2a. Leukocytes show a left-sided asymmetric curve of DNA basal damage (0 Gy control sample) and reach a nearly bell-shaped curve after 4 Gy. To test the sensitivity of the comet assay for subpopulations, samples irradiated with different doses (0, 2, 4 or 8 Gy) were mixed in equal proportions (1:1). Thirty μl of one sample was combined with the next higher irradiation dose level sample. Comparison of means demonstrated highly significant differences in almost all pairs and a trend for cells irradiated with 2 Gy versus 2 + 4 Gy (Table 1). At least two populations were detectable for mixture-samples from 0 to 4 Gy, as indicated by an arrow between both peaks in Figure 2a. A clear detection of different populations in mixture 4 + 8 Gy was impossible due to superimposing caused by a low ascent of both singular curves for 4 and 8 Gy. Regarding histograms, mean differences and standard deviations, we decided to use 4 Gy as optimal radiation dose, enabling best differentiation of DNA damage from baseline and allowing detection of subpopulations (Figure 2a/b).

**Table 1:** Discrimination of heterogeneity with the alkaline comet assay

			**Paired comparison**		
**Sample**	**%Tail DNA**	**SD**	**Compared pair**	**F-value**	**p-value**
0 Gy	6.2	7.6			
0 + 2 Gy	9.7	6.5	0 Gy / 0 + 2 Gy	24.03	<0.001
2 Gy	15.5	6.9	0 + 2 Gy / 2 Gy	74.31	<0.001
2 + 4 Gy	16.9	8.9	2 Gy / 2 + 4 Gy	3.220	0.073
4 Gy	20.7	8.1	2 + 4 Gy / 4 Gy	20.36	<0.001
4 + 8 Gy	30.1	12.3	4 Gy / 4 + 8 Gy	80.78	<0.001
8 Gy	43.1	14.4	4 + 8 Gy / 8 Gy	94.78	<0.001

Leukocytes (200 leukocytes/sample of one donor) were irradiated with increasing doses (0-8 Gy) and mixed in equal proportions with the next higher irradiation dose level sample. Comparison of mean DNA damage in %Tail DNA for samples treated with 0, 2, 4 or 8 Gy and mixtures (0 and 2 Gy, 2 and 4 Gy, 4 and 8 Gy) revealed significant differences for almost all pairs and a trend for cells irradiated with 2 Gy vs. 2 + 4 Gy.

*Abbreviations:* SD = standard deviation, p-value = probability value, F-value.

### Heterogeneity measured by variance

**Figure 3:**
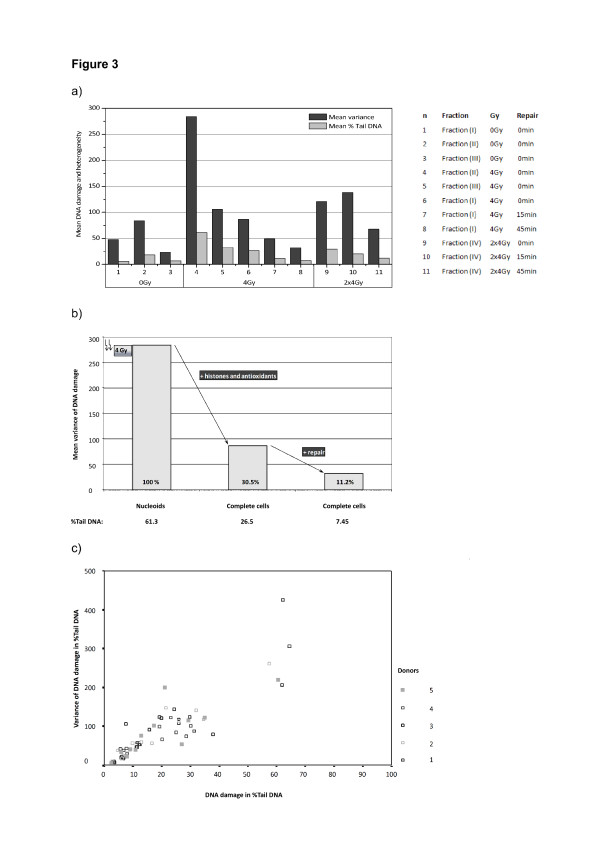
**DNA damage and heterogeneity.a)** Mean variance and DNA damage in %Tail DNA measured after different experimental conditions (see Figure 1) were shown^*^b) The reduction of heterogeneity by histones plus antioxidants and subsequent repair after 4 Gy was represented. Both conditions decreased heterogeneity of DNA damage to approximately one third of the initial level (fraction II i.e. pure DNA plus 4 Gy) ^*^. **c)** The positive correlation (Pearson and Bravais’correlation, r = +0.880; p < 0.001) between DNA damage in %Tail DNA and variance of DNA damage was further substantiated by multiple linear regression (R² = 0.771) indicating a 77.1% dependency of variance by changes of DNA damage. The relationship between both parameters was visualised by a scatter graph (12 samples with 5 independent experiments, n = 60) ^*^.^*^200 leukocytes/sample of each donor were measured for calculation of DNA damage in %Tail DNA and variance. Mean variance was calculated from 5 donors.

The experimental schedule was performed as demonstrated in Figure 1 for different DNA-organisations (fraction I-III) or radiation schedules (fraction IV). DNA damage of two-hundred single cells was measured in %Tail DNA and heterogeneity of damage was estimated by variance. Mean DNA damage in %Tail DNA, mean variance (M_Var_) and one-way analysis of variance (ANOVA) were calculated as shown in Table 2, 3 and Figure 3a.

**Table 2:** ANOVA of mean variance

**ANOVA of mean variances**					
**Sample**	**M_Var_**	**Sample**	**M_Var_**	**F-value**	**p-value**
0Gy (I)	47.63	0Gy (II)	83.81	2.493	0.153
0Gy (I)	47.63	0Gy (III)	23.03	2.551	0.149
0Gy (II)	83.81	0Gy (III)	23.03	11.624	0.009
4Gy (I)	86.53	4Gy (II)	284.02	23.365	0.001
4Gy (I)	86.53	4Gy (III)	106.01	1.807	0.216
4Gy (II)	284.02	4Gy (III)	106.01	9.506	0.002
0Gy (I)	47.63	4Gy (I)	86.53	4.328	0.071
0Gy (II)	83.81	4Gy (II)	284.02	21.728	0.002
0Gy (III)	23.03	4Gy (III)	106.01	70.647	<0.001
4Gy (I)	86.53	4Gy+15min (I)	48.96	10.140	0.013
4Gy (I)	86.53	4Gy+45min (I)	31.92	20.575	0.002
4Gy+15min (I)	48.96	4Gy+45min (I)	31.92	9.887	0.014
2x4Gy (IV)	120.51	2x4Gy+15min (IV)	138.36	0.947	0.359
2x4Gy (IV)	120.51	2x4Gy+45min (IV)	67.67	31.585	<0.001
2x4Gy+15min (IV)	138.36	2x4Gy+45min (IV)	67.67	14.608	0.005
2x4Gy (IV)	120.51	4Gy (I)	86.53	6.839	0.031
2x4Gy+15min (IV)	138.36	4Gy+15min (I)	48.96	26.039	0.001
2x4Gy+45min(IV)	67.67	4Gy+45min (I)	31.92	19.795	0.002

Analysis of variance (ANOVA) was performed for the following pairs (1.-6.) of fraction I-IV^*^. (1.) Influence of DNA structure, antioxidants on heterogeneity of DNA damage without irradiation (line 1–3) and (2.) with irradiation (line 4–6). (3.) Influence of different radiation doses (line 7–9), (4.) heterogeneity of samples with increasing repair periods after single (line 10–12) and double fractionated irradiation (line 13–15) and (6.) comparison of single vs. double fractionated irradiation after same repair periods (line 16–18). In brief, heterogeneity was always significantly increased in histone- and antioxidant-depleted cells (fraction II) and after single or double fractionated irradiation but decreased with repair time or addition of antioxidants. Related mean variance (five donors) and mean DNA damage were shown in Figure 3a

*Abbreviations:* F = F-value, p = probability value. M_Var_ = mean variance.

^*^ 200 leukocytes/sample of each donor were measured for calculation of DNA damage in %Tail DNA and variance. Mean variance was calculated from 5 donors.

**Table 3:** Absolute DNA damage in %Tail DNA of fraction I-IV

**n**	**Fraction**	**[Gy]**	**Repair [min]**	**%Tail DNA of five donors**					**Mean %**
				**Donor1**	**Donor2**	**Donor3**	**Donor4**	**Donor5**	**Tail DNA**
1	I	0	0	5.91	5.56	4.60	7.45	5.89	5.88
2	II	0	0	20.19	24.39	16.66	12.38	17.31	18.19
3	III	0	0	6.56	6.68	6.19	6.82	7.83	6.82
4	II	4	0	64.44	62.09	57.45	61.83	60.54	61.27
5	III	4	0	23.02	31.20	34.51	37.96	34.83	32.30
6	I	4	0	24.90	28.56	25.95	25.91	27.17	26.50
7	I	4	15	11.45	12.22	12.83	11.11	10.99	11.72
8	I	4	45	7.75	7.89	6.61	6.13	8.89	7.45
9	IV	2x4	0	30.05	29.58	31.94	25.88	29.27	29.34
10	IV	2x4	15	19.11	19.16	21.39	19.92	20.95	20.11
11	IV	2x4	45	12.02	11.45	9.79	15.54	12.90	12.34

Mean DNA damage in %Tail DNA of donor leukocytes for fraction I-IV was represented^*^. The highest levels of basal and initial DNA damages (at 0 and 4 Gy) were detected for nucleoids without antioxidants or histones (fraction II).

*Abbreviations:* n = number, Gy = Gray.

^*^ 200 leukocytes/sample of each donor were measured for calculation of DNA damage in %Tail DNA and variance. Mean variance was calculated from 5 donors.

Comparison of heterogeneity of fraction I-III demonstrated highest variance for fraction II with and without irradiation. However, there was no difference between variance of fraction I and III (line 1–6 of Table 2). Intrafractional comparison of 0 vs. 4 Gy (Table 2, line 7–9) revealed that heterogeneity increased with radiation dose of 4 Gy in all fractions by two- to fivefold reaching significance (fraction II, III) or a trend with p = 0.07 (fraction I). Otherwise, heterogeneity and related DNA-damage decreased significantly with repair time after single irradiation (fraction I, Table 2, line 10–12). The same result was found after double fractionated irradiation with increasing repair time (Table 2, line 13–15), but a significant reduction of heterogeneity required more than 15 min of repair time. When single and double fractionated irradiation (fraction I vs. IV) after same repair periods were compared (Table 2, line 16–18), heterogeneity of double fractionated irradiated samples always remained significantly higher than samples after single irradiation. However, absolute levels of DNA damage were in the same range (29.3 vs. 26.5 %Tail DNA at 2×4 vs. 4 Gy, Table 3). Therefore, a second radiation course was clearly distinguishable from single irradiation by variance while absolute DNA damage was equal.

### Heterogeneity, a multi-component model

The relative and absolute influence of the parameters described above on heterogeneity was illustrated for irradiation with 4 Gy in Figure 3b. Nucleoids of DNA with depleted histones (fraction II) showed a maximum of DNA damage and heterogeneity. Heterogeneity was reduced to about one third in intact cells and addition of repair time led to further significant reduction of heterogeneity.

### Correlation between % tail DNA and variance

We graphically evaluated the direction of mean DNA damage in %Tail DNA and mean variance depending on the conditions described above. Increasing DNA damage in %Tail DNA also increased the mean variance in almost all samples. Pearson and Bravais’ correlation of DNA damage and variance substantiated this finding by a significant positive correlation (Correlation coefficient r = +0.880; p < 0.001, Figure 3c). Multiple linear regression calculated an R² of 0.771 indicating a 77.1% dependency of variance on changes of DNA damage. The linear regression coefficient of mean DNA damage was 4.273 (two-tailed significance level p < 0.001) substantiating that %Tail DNA and variance of DNA damage correlated positively.

## Discussion

We demonstrated a simple method to compare heterogeneity by variance analysis. This study is the first – to our knowledge – to confirm that heterogeneity of DNA damage can be selectively modified and is not a constant biological phenomenon. Our results visualize the wide range of heterogeneity substantiated by considerable in- or decreases of variance induced by biological factors as shown in Table 4 and Figure 3. Olive et al. also attributed most proportion of heterogeneity to biological (and not technical) reasons and explained heterogeneity by different susceptibility of areas to loose supercoiling after receiving DNA damage [16]. Practical implications for radiosensitivity testing in certain situations can be derived: In general, best discrimination of cell lines is possible when there is a large difference in absolute DNA damage and heterogeneity of response is low. If the first condition cannot be met, we recommend evaluating single or double fractionated irradiation and repair phase by ANOVA, because heterogeneity measured as variance generated better distinguishable i.e. significant differences during repair phase (0 vs. 45 min) in contrast to lower changes in absolute DNA damage as shown in Table 2 and 3. Therefore, variance analysis after single/double fractionated irradiation and of repair phase might broaden the spectrum of radiosensitivity assessment and help to discriminate cell lines with the same absolute response rates but different heterogeneity.

**Table 4:** Correlation between %Tail DNA and variance

**Factor**	**Changes of amount of**	
	**%Tail DNA**	**Variance**
Single irradiation vs. no irradiation	↑	↑
Single irradiation and repair	↓	↓
Double irradiation vs. no irradiation	↑	↑
Double irradiation and repair	↓	↓
Double vs. single irradiation (+/- repair)	No change	↑
Loss of antioxidants and chromatin	↑↑	↑↑
Addition of antioxidants	↓	↓

Positive correlation of %Tail DNA and variance according to measurements of Table 2 and 3 were indicated by arrows. The only measurements with no change in damage but increase in heterogeneity were comparisons of single vs. double fractionated irradiation. Therefore, this parameter could potentially work as a differentiator of radiation sensitivity.

*Abbreviations:* ↑↑= very strong increase, ↑=increase, ↓=decrease.

A precondition of our study was a method capable of determining heterogeneity including subpopulations. Comet assay fulfilled this condition, as shown by experiments with differentially irradiated cell mixtures and confirmed by other investigators [17-20]. Furthermore, excellent reproducibility and sensitivity for detection of small drug- or radiation-related damages were substantiated for SCGE [21-25] and led to its application in bio-monitoring studies [13,26,27]. Therefore, this method was chosen to investigate the hypothesis that heterogeneity might be related to DNA-conformation, radical scavenging capacity and treatment.

A potential limitation of this study might be the use of leucocytes i.e. of lymphocytes and granulocytes/monocytes. Regarding initial damages without repair, no relevant differences in radiosensitivity were observed for different blood cells [28]. Otherwise, Banath et al. re-ported about slightly faster repair of blood cells compared to isolated lymphocytes which was partly related to different experimental conditions (additional separation procedure for lymphocytes and irradiation in blood tubes compared to frosted slides in this study) [29]. However, all donors in this study had to fulfil inclusion criteria ensuring that the blood cell count was in the reference range excluding larger differences between blood subpopulations of the donors. Furthermore, we performed intraindividual experiments i.e. one blood sample of the donor was divided in the different fractions (I-IV) as described in Figure 1. Therefore, all samples/fractions of one donor contained the same distribution of blood cells minimising potential bias.

We used a model of nucleoids (i.e. DNA with depleted histones) without antioxidants (fraction II) [14] and a second nucleoid model with antioxidants (fraction III) [15]. Different amounts of chromatin or antioxidants are associated with quantitative and qualitative differences in DNA damage [14,15] and with changes of heterogeneity in this setting. Independent of multiple processes, underlying repair and chromatin interactions such as chromatin remodelling [30], chromatin structure at time of damage [31-33] and many others, we detected a significant reduction of heterogeneity with repair time. This effect partly contributes to a repair-related reduction of absolute DNA damage and due to positive correlation with heterogeneity, variance is also reduced. Furthermore, the rearrangement of chromatin remodelling-complexes itself facilitates nucleotide excision repair [34], explaining an amplification of DNA damage reduction. In addition, the susceptibility to radiation-induced hydroxyl radicals changes due to different radical scavenging property of complexed histones [35].

However, the presence of a high concentration of radical scavengers (fraction III) switched heterogeneity to the same level as complete cells, leading to the conclusion that an increase in antioxidants reduces heterogeneity as well as absolute DNA damage. This was also reported by Tiwari et al. [36]. To better visualize this multi-component model of heterogeneity, we marked modifications of absolute damage in %Tail DNA and heterogeneity with arrows, Table 4. The positive correlation of heterogeneity and DNA response is obvious. However, the only parameter which clearly increased heterogeneity while %Tail DNA persisted was double fractionated irradiation with subsequent repair. Therefore, this parameter might be used to further characterize radiation sensitivity as a factor which is independent of absolute response rate.

Our approach offers several practical implications with regard to optimization of radiosensitivity testing by comet assay. Dose response curves are generally compared by changes of mean %Tail DNA or Tail moment. Statistical evaluations of comet assay data were usually performed with a parametric (t-test) or less powerful non-parametric tests (Wilcoxon-test, Mann-Whithney-U-test and others) [37]. Recently ANOVA was recommended as a statistical analysis strategy by the Pharmaceutical Industry Toxicology Special Interest Group [38]. If heterogeneity of radiation response is regarded as an additional valuable parameter for radiosensitivity, ANOVA has the advantage to integrate this information.

Especially in cell lines with same %Tail DNA levels, different heterogeneity might influence biological behaviour and potentially explain occasional discrepancies between comet assay and response prediction assays such as colony forming unit [39,40] or micronucleus assay [41], while most others found good correlations [42-47] or recommended considering multiple parameters [48]. As cell lines with homogeneous response biologically might differ from cell lines with heterogeneity due to radioresistant subgroups [18], heterogeneity might be a marker for less predictable response due to a non-negligible amount of resistant cells. From this point of view, the biological relevance of heterogeneity is obvious and the here described method of modification and calculation might enhance response prediction.

Therefore, additional parameters such as variance of initial and residual damage and the implementation of a second radiation course could expand the available set of methods for improved prediction of radiation sensitivity by comet assay.

## Conclusions

Heterogeneity of comet assay data measured by variance can be selectively modified by changes of chromatin structure, antioxidant concentration, repair and radiation dose. This finding facilitates optimization of experimental conditions by reducing scatter of comet assay data, potentially allowing improved discrimination of small differences and additional radiobiological characterization of cell lines by the amount of heterogeneity.

## Competing interests

The authors declare that they have no competing interests.

## Authors’ contributions

CS has made substantial contributions to acquisition, analysis and interpretation of data. CL has made statistical evaluation and gave advice regarding statistical models and interpretation of data. JD has been involved in revising the manuscript for important intellectual content and has given final approval of the version to be published. ACM has made substantial contributions to conception, design and interpretation of data and manuscript. All authors read and approved the final manuscript.
